# The Murine Natural Cytotoxic Receptor NKp46/NCR1 Controls TRAIL Protein Expression in NK Cells and ILC1s

**DOI:** 10.1016/j.celrep.2018.03.023

**Published:** 2018-03-27

**Authors:** Sam Sheppard, Iona S. Schuster, Christopher E. Andoniou, Clement Cocita, Thomas Adejumo, Sam K.P. Kung, Joseph C. Sun, Mariapia A. Degli-Esposti, Nadia Guerra

**Affiliations:** 1 Department of Life Sciences, Imperial College London, London SW7 2AZ, UK; 2 Immunology and Virology Program, Centre for Ophthalmology and Visual Science, The University of Western Australia, Crawley, Western Australia, Australia; 3 Centre for Experimental Immunology, Lions Eye Institute, Nedlands, Western Australia, Australia; 4 Medical Research Center, Hammersmith Hospital, London W12 0NN, UK; 5 Department of Immunology, Max Rady College of Medicine, University of Manitoba, Winnipeg R3E 0T5, Manitoba, Canada; 6 Memorial Sloan Kettering Cancer Center, Zuckerman Research Center, 408 East 69th Street, New York, NY 10065, USA

**Keywords:** NK cell, natural killer cell, NKp46, ILC1, TRAIL, IL-15, IL-2

## Abstract

TRAIL is an apoptosis-inducing ligand constitutively expressed on liver-resident type 1 innate lymphoid cells (ILC1s) and a subset of natural killer (NK) cells, where it contributes to NK cell anti-tumor, anti-viral, and immunoregulatory functions. However, the intrinsic pathways involved in TRAIL expression in ILCs remain unclear. Here, we demonstrate that the murine natural cytotoxic receptor mNKp46/NCR1, expressed on ILC1s and NK cells, controls TRAIL protein expression. Using NKp46-deficient mice, we show that ILC1s lack constitutive expression of TRAIL protein and that NK cells activated *in vitro* and *in vivo* fail to upregulate cell surface TRAIL in the absence of NKp46. We show that NKp46 regulates TRAIL expression in a dose-dependent manner and that the reintroduction of NKp46 in mature NK cells deficient for NKp46 is sufficient to restore TRAIL surface expression. These studies uncover a link between NKp46 and TRAIL expression in ILCs with potential implications in pathologies involving NKp46-expressing cells.

## Highlights

• NKp46-deficient mice lack constitutive expression of TRAIL on liver resident ILC1s
• NKp46-deficient splenic NK cells fail to induce cell surface TRAIL upon activation
• NKp46 regulates TRAIL expression at a post-translational level
• Transduction of NKp46 in primary NK cells restores TRAIL surface expression

## Graphical Abstract

**Figure:**
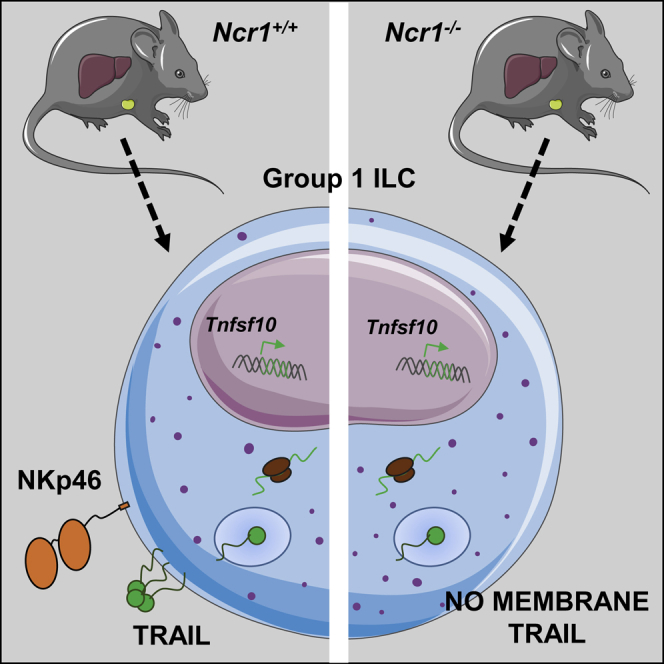

## Teaser

Sheppard et al. find that mice deficient in the activating receptor NCR1/NKp46 (*Ncr1*^*−/−*^*)* fail to express the apoptosis-inducing ligand TRAIL at the surface of group 1 innate lymphoid cells (ILC1s).

## Introduction

Natural killer (NK) cells are innate lymphoid cells able to discriminate and eliminate infected cells and tumor cells because of a large panel of germline-encoded receptors (Biassoni et al., 2001, Lanier, 2005). The stimulatory receptor NKp46 is one of the natural cytotoxic receptors (NCRs) expressed on all NK cells (Pessino et al., 1998). It efficiently triggers the release of cytotoxic granules, cytokines, and chemokines upon binding ligands of viral (Mandelboim et al., 2001), bacterial (Vankayalapati et al., 2002), and cellular origin (Narni-Mancinelli et al., 2017) in addition to unidentified ligands on tumor cells (Cagnano et al., 2008). Human NKp46 and its mouse ortholog NKp46/NCR1 (CD335) (Biassoni et al., 1999) are immunoglobulin (Ig)-like transmembrane glycoproteins. In addition to NK cells, NKp46 is also expressed by type 1 innate lymphoid cells (ILC1s) (Cortez and Colonna, 2016, Zook and Kee, 2016), a subset of group 3 ILCs (Montaldo et al., 2015) and a small subset of T cells. NKp46-deficient mice (*Ncr1*^*gfp/gfp*^) have been widely used to demonstrate the importance of NKp46 in the control of microbial infection (Gazit et al., 2006) and tumor development by NK cells (Glasner et al., 2012) as well as in contributing to type 1 diabetes (Gur et al., 2010).

When activated, NK cell lytic activity is mainly mediated via exocytosis of cytotoxic granules containing a payload predominantly made up of perforin and granzymes. Other pathways include the engagement of death receptors via membrane-bound or soluble proteins that belong to the tumor necrosis factor (TNF) family of cytokines (Falschlehner et al., 2009). The tumor necrosis factor-related apoptosis-inducing ligand (TRAIL/Apo2L) is a type II transmembrane protein (Wiley et al., 1995) constitutively expressed on liver-resident NK cells in humans (Stegmann et al., 2016) and in mice (Peng and Tian, 2017, Takeda et al., 2005, Yokoyama et al., 2013), a population of cells that has recently been categorized as ILC1s (Cortez and Colonna, 2016, Jiao et al., 2016). In addition to TRAIL, the transcription factors T-bet and Eomesodermin (Eomes) are commonly used to identify resident NK cells in the mouse (Daussy et al., 2014, Gordon et al., 2012) and human liver tissue (Cuff et al., 2016, Harmon et al., 2016). Other markers include chemokine receptors (Stegmann et al., 2016) and integrins of the CD49 antigen-like family (Aw Yeang et al., 2017, Daussy et al., 2014, Gordon et al., 2012), showing similarities between mouse- and human-resident NK cells described in healthy livers (Marquardt et al., 2015). These tissue-resident ILC1s have recently been shown to represent a major early source of interferon γ (IFN-γ), making them important first responders to viral infection (Weizman et al., 2017)

Human and mouse TRAIL engage receptors that possess a death domain and induce caspase 8-mediated apoptosis, including TRAIL-R1/DR4 in humans and TRAIL-R2/DR5 in both species (Walczak et al., 1997). Other receptors include TRAIL-R3 and TRAIL-R4—considered a decoy receptor because of the lack of or incomplete death domain—and the soluble protein Osteoprotegerin, none of which promote cell death (Degli-Esposti, 1999). TRAIL is a well-established player in anti-tumor immunity, potently clearing TRAIL-R-expressing tumors without affecting normal primary tissue (Walczak et al., 1999). Indeed, several reports established that TRAIL is involved in NK cell-mediated rejection of transplanted tumors expressing TRAIL-R (Smyth et al., 2001, Takeda et al., 2001), chemically induced sarcoma (Cretney et al., 2002), and liver metastases (Cretney et al., 2002, Seki et al., 2003, Smyth et al., 2001) as well as hematological malignancies (Zerafa et al., 2005).

An important and increasingly recognized function of TRAIL is its involvement in the regulatory function of NK cells (Hayakawa et al., 2004), especially in contexts of virally induced chronic inflammation (Maini and Peppa, 2013, Schuster et al., 2016). In the well-studied model of mouse cytomegalovirus (MCMV) infection, NK cells can limit the function and antiviral T cell responses via elimination of MCMV-infected dendritic cells (Andrews et al., 2010) and CD4^+^ T cells (Schuster et al., 2014). Inflammatory cytokines such as IFNs (Smyth et al., 2001, Stegmann et al., 2010), interleukin-26 (IL-26) (Miot et al., 2015), and IL-15 and IL-2 (Kayagaki et al., 1999) have been found to induce TRAIL expression on NK cells; however, the intrinsic pathways regulating TRAIL expression have not been defined. Using the NKp46-deficient mouse strain *Ncr1*^*gfp/gfp*^ (designated *Ncr1*^*−/−*^ hereafter), the present study uncovers a link between TRAIL and NKp46, showing that NKp46 is necessary and sufficient for TRAIL surface expression in ILC1s and NK cells.

## Results

### NKp46 Is Necessary for TRAIL Surface Expression on NK Cells and ILC1s

**Figure 1:**
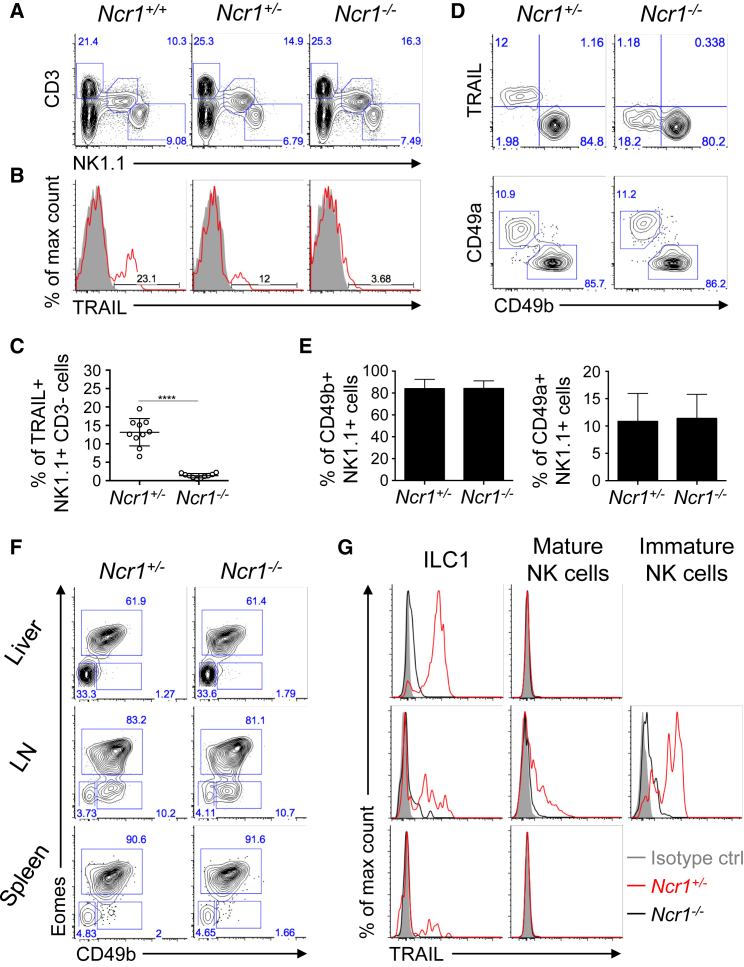
ILC1s Lack TRAIL Expression in NKp46-Deficient Mice (A) Representative flow cytometry plots showing frequencies of T cells (CD3^+^ NK1.1^−^), NKT cells (CD3^+^ NK1.1^+^), and NK cells (CD3^−^ NK1.1^+^) in the livers of naive wild-type mice, *Ncr1*^*−/−*^ mice, or heterozygous *Ncr1*^*+/−*^ mice. (B and C) Representative flow cytometry histograms (B) and average percentage (± SD) (C) of TRAIL^+^ group1 ILCs detected in the livers of *Ncr1*^*+/−*^ and *Ncr1*^*−/−*^ mice. (D and E) Representative flow cytometry plots of TRAIL, CD49b/DX5, and CD49a expression on hepatic group 1 innate lymphoid cells (CD3^−^ NK1.1^+^) from naive *Ncr1*^*+/−*^ and *Ncr1*^*−/−*^ mice (D) and average percentage (± SD) of CD49b/DX5^+^ NK cells (E, left) and CD49a^+^ NK cells (E, right) as described in (D). (F) Representative flow cytometry plots of the gating strategy used to distinguish (CD3^−^ NK1.1^+^) ILC subsets: mature NK cells (CD49b^+^Eomes^+^) from immature NK cells (CD49b^+^Eomes^−^) and ILC1s (CD49b^−^ Eomes^−^) in liver, lymph node (LN), and spleen tissues harvested from *Ncr1*^*+/−*^ and *Ncr1*^*−/−*^ mice. (G) Representative flow cytometry histograms of TRAIL expression on the cell subsets defined in (F). Data are representative of 2–4 experiments, each with 2–5 mice per group. ^∗∗∗∗^p < 0.0001 (unpaired t test).

While characterizing different subsets of liver NK cells in resting NKp46-deficient mice (*Ncr1*^*−/−*^ ) (Sheppard et al., 2013), we discovered that CD3^−^ NK1.1^+^ NK cells lacked TRAIL surface expression, in contrast with their wild-type (*Ncr1*^*+/+*^) and heterozygote (*Ncr1*^*+/−*^) littermates (Figures 1A–1C). To distinguish liver ILC1s, also known as resident TRAIL^+^ NK cells, from conventional mature NK cells, we used DX5 (CD49b) and CD49a markers. We observed similar proportions of liver ILC1s (CD49b/DX5^−^ CD49a^+^) and mature NK cells (CD49b/DX5^+^CD49a^−^) in both strains (Figures 1D and 1E; Sheppard et al., 2013). ILC1s were further identified as DX5^−^ Eomes^−^ in the livers of *Ncr1*^*+/+*^ mice, where they represented the main population of TRAIL-expressing cells, as expected (Figures 1F and 1G). However, in the *Ncr1*^*−/−*^ mouse, TRAIL was virtually absent from liver ILC1s that were present at normal frequency (Figures 1F and 1G). Similarly, TRAIL was absent from small populations of ILC1s detected in the spleen and lymph nodes of *Ncr1*^*−/−*^ mice as well as from mature and immature NK cells present in the lymph nodes (Figures 1F and 1G). Hence, the absence of TRAIL expression in the *Ncr1*^*−/−*^ mouse is not due to a defect in the differentiation of NK cells and ILC1s but a direct consequence of the lack of NKp46.

### NKp46 Positively Regulates TRAIL Induction *In Vivo*

**Figure 2:**
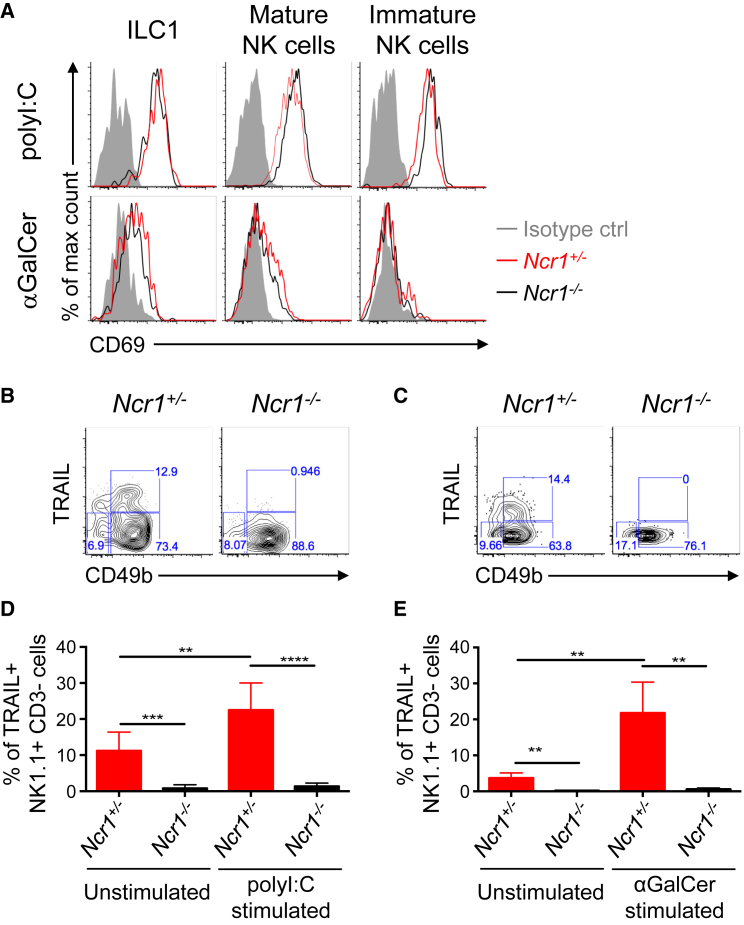
NKp46-Deficient NK Cells and ILC1s Fail to Upregulate TRAIL upon *In Vivo* Activation (A) Representative flow histograms of CD69 expression on ILC1s and mature and immature NK cells isolated from *Ncr1*^*+/−*^ and *Ncr1*^*−/−*^ mice stimulated with poly(I:C) for 24 hr (top) and the CD1d ligand α-galactosylceramide (α-GalCer) for 9 days (bottom). (B and C) Representative flow cytometry plots showing expression of TRAIL and CD49b/Dx5 expression on (CD3^+^ NK1.1^+^) cells isolated from *Ncr1*^*+/−*^ and *Ncr1*^*−/−*^ mice stimulated with poly(I:C) (LN) (B) and α-GalCer (spleen) (C) as described above. (D and E) Bar graph representing the average percentage (± SD) of TRAIL^+^ NK cells (CD3^−^ NK1.1^+^) isolated from *Ncr1*^*+/−*^ and *Ncr1*^*−/−*^ mice left unstimulated (PBS) or stimulated as described above with poly(I:C) (LN) (D) and α-GalCer (spleen) (E). Data are representative of 2–4 experiments, each with 2–5 mice per group. The p values were measured by unpaired t test. See also Figure S1.

To investigate TRAIL induction *in vivo*, we injected *Ncr1*^+/+^ and *Ncr1*^−/−^ mice with poly(I:C) and α-galactosylceramide (α–GalCer), 2 immunogenic compounds known to upregulate TRAIL on NK cells via induction of proinflammatory cytokines (Smyth et al., 2001, Takeda et al., 2005, Tu et al., 2011). NK cells and ILC1s retrieved from the lymph nodes of poly(I:C)-injected *Ncr1*^+/+^ and *Ncr1*^−/−^ mice showed similar expression profiles of the activation marker CD69, indicating comparable activation levels (Figure 2A). Nonetheless, in the absence of NKp46, mature NK cells and ILC1s analyzed from the lymph nodes of *Ncr1*^−/−^ mice were unable to induce TRAIL post-stimulation with poly(I:C) (Figures 2B and 2D). We obtained similar results upon α-GalCer stimulation, where splenic NK cells in *Ncr1*^−/−^ mice were unable to induce TRAIL cell surface expression (Figures 2C and 2E). We have previously demonstrated that NKp46-deficient NK cells produce comparable amounts of IFN-γ upon activation with IL-2 and IL12 + IL-18 and upon triggering of the activating receptors NK1.1, Ly49D, and NKG2D to that produced by wild-type NK cells (Sheppard et al., 2013). In addition, expression of the IFN-γ receptor (CD119) is equivalent in NK cells from *Ncr1*^−/−^ and *Ncr1*^+/+^ mice (Figure S1). Collectively, these data indicate that the inability of NK cells to induce TRAIL in the absence of NKp46 is not due to a defect in NK cell activation.

### IL-2 and IL-15 Fail to Upregulate TRAIL on Mature *Ncr1*^−/−^ NK Cells

**Figure 3:**
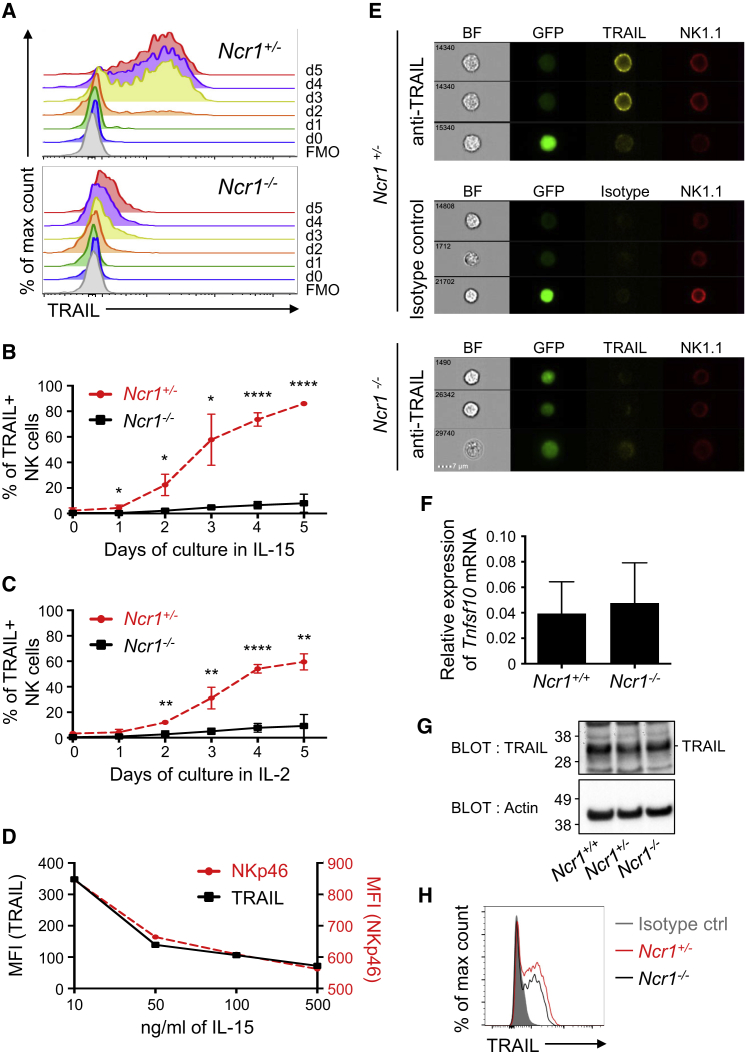
IL-2 and IL-15 Fail to Induce TRAIL Protein Expression at the Membrane of NKp46-Deficient NK Cells (A) Representative flow histograms of TRAIL induction on IL-15-activated splenic NK cells (CD3^−^ NK1.1^+^) isolated from *Ncr1*^*+/−*^ (top) and *Ncr1*^*−/−*^ (bottom) mice (5 day culture in IL-15, 50 ng/mL). The negative control is depicted as fluorescence minus one (FMO). (B and C) Average percentage (± SD) of TRAIL^+^ NK cells generated over 5 days of culture in the presence of IL-15 (50 ng/mL) (n = 3 mice/genotype) (B) and IL-2 (50 U/ml) (n = 3 mouse/genotype) (C). Values represent means ± SD. Statistical significance was measured via unpaired Mann-Whitney test). (D) Mean fluorescence intensity of TRAIL and NKp46 co-expressed on splenic NK cells shown on day 5 for various concentrations of IL-15 as indicated in the plot. The data in (A)–(D) are representative of 4 or more experiments. (E) Representative confocal images obtained by ImageStream analysis of IL-15-activated NK cells isolated from *Ncr1*^*−/−*^*(Ncr1*^*gfp/gfp*^*)* and *Ncr1*^*+/−*^*(Ncr1*^*gfp/+*^*)* mice that express endogenous GFP. Staining with antibodies specific for NK1.1 and TRAIL or isotype phycoerythrin (PE) control is shown, as well as bright-field (BF) images. Zombie dye was used to gate out dead cells. Three cells representative of at least 480 events acquired (GFP^+^ NK cells) per condition are shown and are representative of 3 independent experiments. The scale bar represents 7 μm. (F) Bar graph depicting the relative average expression (± SD) of *Tnfsf10* mRNA in IL15-activated splenic NK cells isolated from *Ncr1*^*+/−*^ and *Ncr1*^*−/−*^ mice (5 days culture in IL-15, 50 ng/mL). Data are a pool of 3 mice per group combined from 1–2 experiments. (G) Western blot analysis of the total TRAIL protein expressed in *Ncr1*^*+/+*^, *Ncr1*^*+/−*^, and *Ncr1*^*−/−*^ NK cells upon activation (5 day culture in IL-2, 1,000 U/mL). Data are representative of 2 independent experiments. Actin was used as a reference. (H) Representative flow histogram of TRAIL intracellular staining or isotype control (shaded gray) of IL-2-activated splenic NK cells isolated from *Ncr1*^*+/−*^ (red line) and *Ncr1*^*−/−*^ (black) mice. Data are representative of 2 independent experiments. See also Figures S2 and S3.

Mature splenic NK cells in wild-type mice do not express TRAIL unless activated in culture in the presence of IL-2 (Tu et al., 2011) or IL-15 (Kayagaki et al., 1999, Zamai et al., 1998). To assess the kinetics of TRAIL induction on *in vitro*-activated NK cells, we cultured splenic NK cells isolated from *Ncr1*^+/+^ and *Ncr1*^−/−^ mice for 5 days in the presence of IL-15 or IL-2. A small fraction of NKp46-sufficient NK cells expressed high levels of TRAIL on day 2 that progressively increased over time (Figures 3A–3C). However, NK cells deficient in NKp46 failed to induce significant levels of cell surface TRAIL after 5 days of culture with IL-15 (Figures 3A and 3B) or with IL-2 (Figure 3C). NK cells from *Ncr1*^+/+^ and *Ncr1*^−/−^ mice expressed equivalent levels of the IL-2 receptor α chain (CD25) and the β (CD122) and γ chains (CD132) shared by the IL-2 and IL-15 receptors (Figure S1). Thus, the differences in the induction of TRAIL expression were not the result of differential signaling through these receptors.

Interestingly, we observed a positive correlation between NKp46 and TRAIL expression in *Ncr1*^+/+^ mice, dependent on the concentration of IL-15 present in the culture. Low levels of IL-15 (10 ng/mL) induced high levels of NKp46 and TRAIL that diminished with increasing concentrations of IL-15 (Figure 3D).

Because a small fraction of NKp46-deficient NK cells seems to be weakly positive for TRAIL when assessed via flow cytometry (shoulder positivity), we employed ImageStream to visualize TRAIL at the cell membrane, using NK1.1 expression as positive control. Figure 3E shows that the faint TRAIL staining seen in NKp46-deficient NK cells (bottom) was similar to the signal detected by isotype staining (center) and significantly lower than TRAIL^low^ and TRAIL^hi^ staining in NK cells from wild-type mice (top) (Figure S2). We conclude that TRAIL is mainly absent from NKp46-deficient NK cells, although we cannot totally exclude that a very low amount of TRAIL spontaneously reaches the surface of NKp46-deficient NK cells.

To gain further insight into the stage of TRAIL regulation by NKp46, we quantified the amount of *Tnfsf10* transcripts coding for TRAIL in sorted NK cells cultured in the presence of IL-15 for 5 days. Similar levels of *Tnfsf10* mRNA were detected in NK cells from *Ncr1*^+/+^ and *Ncr1*^−/−^ mice, indicating that NKp46 does not control gene transcription or the stability of *Tnfsf10* mRNA (Figure 3F). This was further shown in liver ILC1s where equal amounts of *Tnfsf10* transcripts were detected in both strains (Figure S3). Western blotting of TRAIL in activated NK cells revealed that the protein was expressed in the presence or absence of NKp46 (Figure 3G), which was confirmed by intracellular detection of TRAIL in NKp46-deficient NK cells (Figure 3H). Collectively, these data show that NKp46 controls TRAIL protein expression at a post-translational level by affecting its trafficking to the membrane.

### NKp46 Is Sufficient to Restore TRAIL Surface Expression in *Ncr1*^−/−^ NK Cells

**Figure 4:**
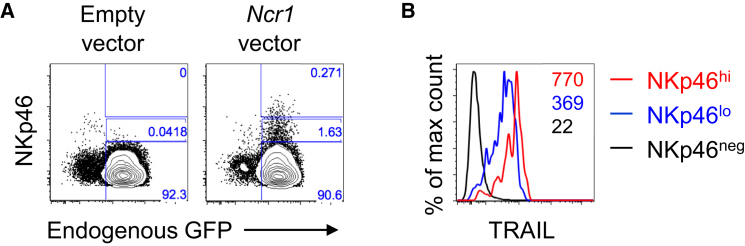
Reintroduction of NKp46 in NKp46-Deficient Cells Restores TRAIL Membrane Expression (A) Representative flow plots of NKp46 expression on *Ncr1*^*−/−*^ mice (*gfp* knockin) NK cells transduced with an *Ncr1-*expressing vector or empty vector and identified via expression of endogenous GFP. Co-staining for NKp46 expression is shown on the y axis. Percentages of NK cells showing no, low, or high expression of NKp46 are indicated on each plot. (B) Representative flow histograms of TRAIL expression on NKp46^high^, NKp46^low^, and NKp46^neg.^ NK cells per gating shown in (A). Data are representative of 3 experiments, each with 1–2 mice per group.

To determine whether NKp46 is sufficient to induce TRAIL surface expression, we transduced primary NK cells isolated from *Ncr1*^−/−^ mice with an Ncr1-expressing vector. The reintroduction of NKp46 restored TRAIL expression in NKp46-deficient NK cells (Figures 4A and 4B). Remarkably, the level of TRAIL and NKp46 expression after transfection positively correlated with the NKp46^low^ NK subset expressing lower levels of TRAIL compared with the NKp46^hi^ subset (Figure 4B). This result is consistent with those obtained using IL-15-activated NK cells (Figure 3D). In conclusion, our data show that NKp46 is sufficient to control TRAIL surface expression in a dose-dependent manner.

## Discussion

In this study, we describe an unexpected link between murine NKp46 and the death-inducing ligand TRAIL, proteins that are coexpressed by ILC1s and NK cells. Analysis of the NKp46-deficient mouse revealed that ILC1s and small subsets of immature NK cells lack TRAIL membrane expression at steady state in an otherwise normal immune context (Sheppard et al., 2013). These data indicate that impaired TRAIL expression in the *Ncr1*^*−/−*^ mouse is caused by the lack of NKp46 and not by developmental defects in the ILC compartment. In addition, in the absence of NKp46, mature NK cells activated *in vitro* and *in vivo* fail to upregulate cell surface TRAIL unless NKp46 is transduced. We demonstrate that NKp46 is necessary for TRAIL surface expression and that this likely involves a cell-intrinsic regulatory mechanism.

This phenotype raises several key questions with regards to the mechanism(s) involved. First, how is NKp46 controlling TRAIL expression? NKp46 likely affects TRAIL trafficking to the membrane because we detected a comparable amount of transcripts encoding TRAIL and cytosolic protein in NKp46-deficient and -sufficient NK cells. Possible mechanisms include a requirement for NKp46 to release TRAIL from cytoplasmic vesicles and/or to act as a chaperone for TRAIL localization to the plasma membrane. Co-localization studies of TRAIL and NKp46 via Förster resonance energy transfer imaging are currently investigating TRAIL trafficking and putative association with NKp46. Second, is NKp46 ligand-induced downstream signaling required for TRAIL cell surface expression in group 1 innate lymphoid cells? If so, does it involve the engagement of NKp46 via membrane and/or intracellular ligands? The transmembrane region of NKp46 is critical for binding the adaptor molecules FcεRIγ and CD3ζ (Westgaard et al., 2004). Studies examining TRAIL expression in NK cells selectively modified in this region and thus defective in NKp46 signaling will provide critical mechanistic insights. Moreover, the *Noé* mouse is a valuable model that might help address the requirement for NKp46 to bind extracellular ligands because the mouse has impaired NKp46 surface expression (Narni-Mancinelli et al., 2012) but retains cytoplasmic NKp46 (Glasner et al., 2015, Narni-Mancinelli et al., 2012). Additional questions arise from the fact that other cell types that do not express NKp46, including monocytes (Ellis et al., 2015) and T cells (Ishikawa et al., 2005), can exhibit cell surface TRAIL expression upon infection, findings that imply that NKp46-mediated control of TRAIL may be specific to the ILC compartment.

With regards to the biological relevance of TRAIL regulation by NKp46, both proteins are known players in anti-tumor responses (Koch et al., 2013, Seki et al., 2003, Walczak et al., 1999); hence, the concomitant expression of NKp46 and TRAIL is likely to potentiate NK cell direct killing activity against tumors bearing NKp46 ligands and TRAIL receptors. Also, TRAIL/TRAIL-R is a critical axis of immunoregulation by NK cells upon persistent infection (Schuster et al., 2014). The constitutive expression of TRAIL on resident hepatic NK cells and upregulation via inflammatory cytokines likely contribute to maintaining an immunosuppressive environment characteristic of the liver tissue and avoiding hepatitis upon prolonged cytokine exposure. Peppa et al. (2013) previously demonstrated that TRAIL^+^ NK cells contribute to persistent infection and liver damage by eliminating hepatitis B virus (HBV)-specific CD8^+^ T cells that upregulate TRAIL-R2, thereby becoming susceptible to TRAIL-mediated apoptosis (Peppa et al., 2013). Recently, Yoshioka et al. (2017) observed a higher frequency of NKp46 in HBV-infected patients with a high viral DNA titer compared with healthy subjects, which correlated with high levels of alanine transaminase (ALT), a hallmark of liver damage (Yoshioka et al., 2017). The authors showed that a subset of NKp46^hi^NKG2A^hi^ NK cells displays an elevated level of TRAIL mRNA and a high cytotoxic potential against activated T cells *in vitro* (Yoshioka et al., 2017). Hence, it is plausible that NKp46 and TRAIL act in concert to target TRAIL-R-expressing immune cells upon persistent inflammation in HBV-infected patients. Similarly, NKp46-deficient mice were used to demonstrate that NK cells can attenuate liver fibrosis via NKp46-mediated killing of hepatic stellate cells (HSCs) that express NKp46 ligands (Gur et al., 2011); TRAIL could potentially be involved in the protective role of NK cells in this model.

NK cells can also participate in tissue damage in the context of persistent inflammation. NK cells contribute to TRAIL-mediated apoptosis of hepatocytes isolated from HBV-infected individuals, suggesting that TRAIL^+^ NK cells have the potential to promote liver damage (Dunn et al., 2007). Similarly, NK cells have been involved in the pathogenesis of autoimmune diseases such as type 1 diabetes. There is a high frequency of activated NKp46^+^ NK cells in diabetic patients (Wang et al., 2015), and NKp46 ligands are detected on human and mouse pancreatic β cells, with the ability to induce NKp46-mediated NK cell degranulation (Gur et al., 2010). Compared with the NKp46-sufficient mouse, NKp46-deficient mice developed low-dose-of-streptozotocin (LDST)-induced type 1 (T1) diabetes at a lower frequency and displayed lower blood glucose levels, demonstrating the role of NKp46 in the development of diabetes (Gur et al., 2010). TRAIL could be involved in the NKp46-mediated NK cell response in this model. Indeed, it is known that the normal human pancreas exhibits a high amount of TRAIL-R under chronic inflammatory conditions such as chronic pancreatitis (Hasel et al., 2003); hence, NK cells could be deleterious via both NKp46/NKp46 ligands and TRAIL/TRAIL-R interactions.

In conclusion, several phenotypes based on studies of the NKp46-deficient mouse may actually arise because of the concomitant lack of TRAIL and NKp46 expression. The relationship between NKp46 and TRAIL described here should be taken into consideration when reflecting on past studies of the *Ncr1*^*−/−*^ mouse and on future studies addressing NKp46 and TRAIL functions.

## Experimental Procedures

Further details and an outline of resources used in this work can be found in the Supplemental Information.

### Animals

*Ncr1*^*gfp/gfp*^ mice (RRID:IMSR_JAX:022739), kindly provided by Prof. Ofer Mandelboim (Gazit et al., 2006), were bred and maintained in the animal facility at Imperial College London in a specific pathogen-free environment. Work was carried out in compliance with the British Home Office Animals Scientific Procedures Act 1986 (PPL70/7129).

### NK Cell Isolation, Activation, and Transduction

NK cells were activated *in vitro* in complete RPMI medium supplemented with IL-2 or IL-15 as indicated in the figure legends. 2 μg of α-GalCer (intraperitoneally [i.p.]) or 100 μg of poly(I:C) was used to activate NK cells *in vivo.* 293T cells were transfected with the vesicular stomatitis virus G protein (VSV-G)-pseudotyped lentiviral vector SIN18-RhMLV-Cppt-2E (Tran and Kung, 2007) containing *GFP* or *Ncr1* using TransIT-293 (Mirus). NK cells that had been cultured for 3 days in 1,000 U/mL IL-2 were transduced with viral particles containing supernatant and assessed for TRAIL and NKp46 expression by flow cytometry.

### RNA Isolation and Real-Time qPCR

Reverse-transcribed cDNA was amplified using the TaqMan PreAmp Master Mix Kit prior to real-time qPCR (TaqMan system, Applied Biosystems). All values were normalized to *Gapdh* expression.

### Western Blotting

Total cell lysate transferred to a polyvinylidene fluoride (PVDF) membrane was immunoblotted with either anti-TRAIL or anti-actin antibodies prior blot analysis (Image Lab software, Bio-Rad).

### ImageStream

Primary splenocytes were stained for the indicated markers and analyzed on ImageStreamX Mark II (Amnis, USA) and analyzed using IDEAS software.

### Statistical Analyses

Two-tailed unpaired Student’s t test and Mann-Whitney tests were applied (as indicated in the figure legends) when appropriate (GraphPad). Differences at p ≤ 0.05 were considered significant: ^∗^p ≤ 0.05, ^∗∗^p ≤ 0.01, ^∗∗∗^p ≤ 0.001, ^∗∗∗∗^p ≤ 0.0001.

## Supplemental Information

Document S1. STAR Methods and Figures S1–S3

Document S2. Article plus Supplemental Information
