# Supplementary material for: The Murine Natural Cytotoxic Receptor NKp46/NCR1 Controls TRAIL Protein Expression in NK Cells and ILC1s
Source: Cell Rep. 2018 Mar 27;22(13):3385–92. doi: 10.1016/j.celrep.2018.03.023 (PMC5896200; doi:10.1016/j.celrep.2018.03.023)
Supplement: Document S1. STAR Methods and Figures S1–S3 [file mmc1.pdf]

Cell Reports, Volume *22*

Supplemental Information

The Murine Natural Cytotoxic Receptor NKp46/NCR1 Controls TRAIL Protein Expression in NK Cells and ILC1s

Sam Sheppard, Iona S. Schuster, Christopher E. Andoniou, Clement Cocita, Thomas Adejumo, Sam K.P. Kung, Joseph C. Sun, Mariapia A. Degli-Esposti, and Nadia Guerra

SUPPLEMENTAL FIGURES

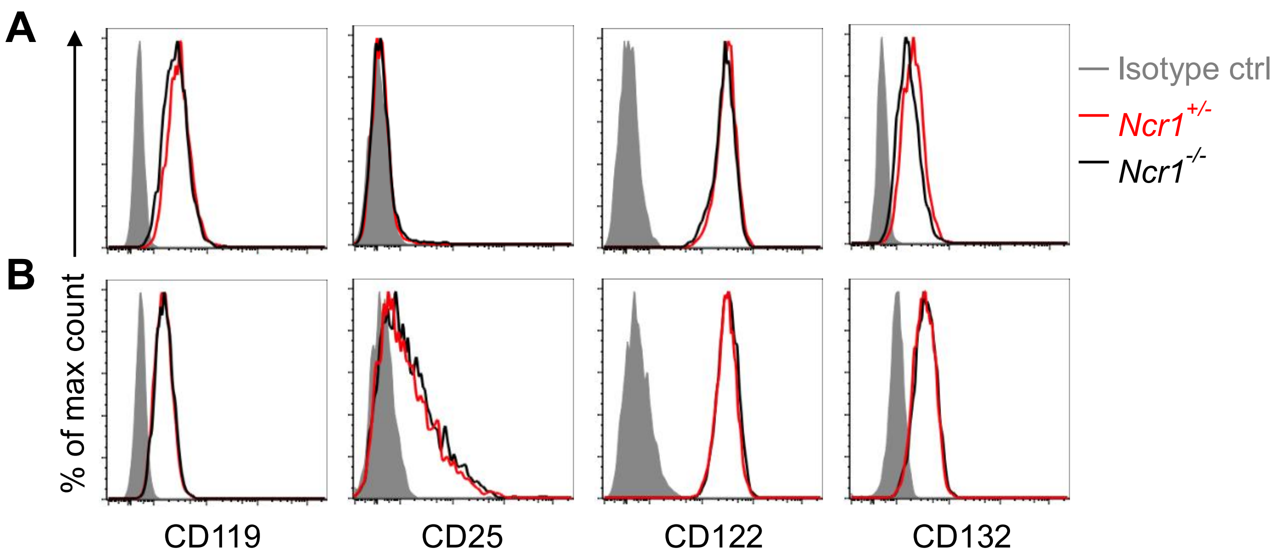

Figure S1. Expression of IFN-γ receptor1, IL-2 and IL-15 receptors are comparable in NKp46-deficient and –sufficient mice. Related to Figure 2. Representative flow histograms depicting the expression of IFN-γ receptor1 (CD119), alpha (CD25), beta (CD122) and gamma chains (CD132) of the IL-2 and IL-15 receptors on *Ncr1*$^{-/-}$ and *Ncr1*$^{+/+}$ NK cells on naïve liver resident NK cells (A) and IL-2 activated NK cells (B). Data are representative of at least 2 independent experiments.

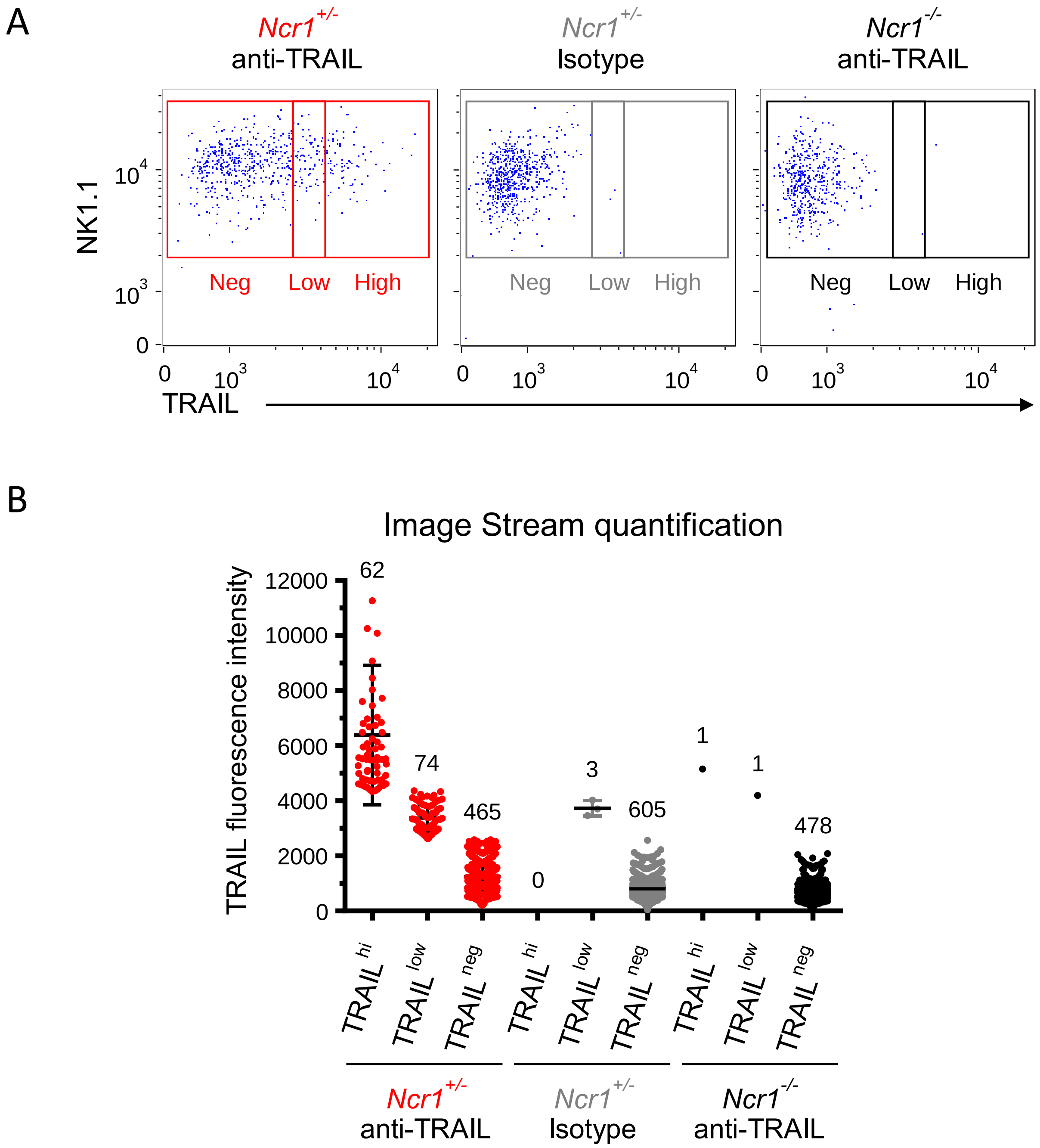

Figure S2. NKp46 is necessary for TRAIL surface expression on NK cells. Related to Figure 3. (A) Representative flow plot of TRAIL and NK1.1 expression (gated on live GFP+ cells) on Il-15 activated NK cells isolated from *Ncr1*$^{+/-}$ and *Ncr1*$^{-/-}$ mice. Data acquired on Image Stream analyser are displayed as TRAIL^{neg}, TRAIL^{low} and TRAIL^{hi} subsets; plots show anti-TRAIL and isotype staining condition. (B)Quantification of TRAIL fluorescence intensity in each subset described in A. Data shown are representative of 3 independent experiments including one mouse per genotype. Numbers indicate the quantity of events (live NK cells) acquired for each subset.

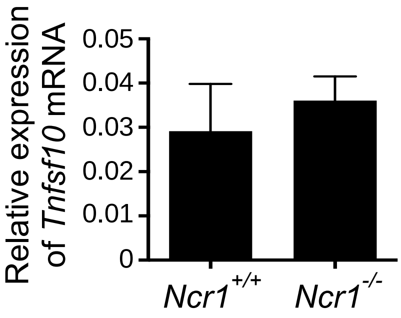

Figure S3. ***Tnfsf10*** **mRNA expression in ILC1.** Related to Figure 3. Bar graph depicts the average expression (± SD) of *Tnfsf10* mRNA (the gene coding for TRAIL) relative to *GAPDH mRNA* in liver resident NK cells ($CD3^-$ $NK1.1^+$) isolated from *Ncr1*$^{+/-}$ and *Ncr1*$^{-/-}$ mice. Data are a pool of 3 mice per group combined from 1-2 experiments.

SUPPLEMENTAL EXPERIMENTAL PROCEDURES

CONTACT FOR REAGENTS AND RESOURCES SHARING

Further information and requests for resources and reagents should be directed to and will be fulfilled by the Lead Contact, Nadia Guerra (n.guerra@imperial.ac.uk).

METHOD DETAILS

Tissue dissociation and flow cytometry

Tissues were dissociated through 100 µm cell strainers in PBS with 3 % bovine serum albumin (BSA). Hepatocytes were removed by centrifugation on a 35 % percoll gradient at 700 xg at 21 °C for 12 min. Leukocytes present in the pellet were resuspended in red blood cell lysis buffer (0.15 M NH_4Cl, 0.1 mM $KHCO_3$, 0.1 mM Na_2-EDTA in water; pH 7.2) for 1 min, washed and resuspended in PBS 3 % BSA. Cell suspensions were incubated with anti-mouse CD16/CD32 (Becton Dickinson - BD - USA) to block Fc receptors, and Fixable Viability Dye eFluor 506 (eBioscience - San Diego, CA, USA). Cells were then stained with a cocktail of directly conjugated and biotinylated mAbs for 30 min at 4 ºC followed by Qdot 605 conjugated streptavidin (Life Technologies, USA) to reveal biotinylated antibodies. For intracellular staining, cells were fixed and permeabilized for 30 min at 4 ºC using the Cytofix/Cytoperm kit (BD Biosciences, UK) or with a transcription factor staining buffer set from eBioscience (ThermoFisher Scientific, UK). Then, cells were washed in PermWash buffer (Becton Dickinson, BD, USA) or Permeabilization buffer (eBioscience, CA, USA). Cells were then stained with a cocktail of directly conjugated and biotinylated mAbs for 30 min at 4 ºC followed by Qdot 605 conjugated streptavidin (Life Technologies, USA) to reveal biotinylated antibodies. For intranuclear anti-Eomes staining, cells were fixed and permeabilized for 30 min at 4 ºC using the transcription factor staining buffer set from eBioscience (San Diego, CA, USA). Then, cells were washed in Permeabilization buffer (eBioscience, CA, USA) and stained with directly conjugated anti-Eomes mAbs in the permeabilization buffer for 30 min at 4 ºC, washed twice in the permeabilization buffer and resuspended in PBS 3 % BSA. For intracellular anti-TRAIL staining, cells were fixed overnight using 0.1 % PFA 3 % BSA solution and permeabilized the next day for 30 min at 4 ºC using a 1 % TWEEN 3 % BSA solution. Then, cells were stained with directly conjugated anti-TRAIL mAbs in 1 % TWEEN 3 % BSA for 30 min at 4 ºC, washed twice in 1 % TWEEN 3 % BSA and resuspended in PBS 3 % BSA. The relevant fluorescence-minus-one labelling conditions including the appropriate isotype mAb were used as controls. All samples were acquired on an LSR Fortessa flow cytometer (BD) and analyzed with FlowJo version 9.3.1 or above (TreeStar, Ashland, OR, USA).

***In vivo* and *in vitro* NK cell culture**

In vivo activation with α–GalCer (Avanti Polar Lipids, Inc. Alabama) was performed via i.p. injection of 2 µg in *$Ncr1^{gfp/gfp}$ and $Ncr1^{gfp/+}$* mice 9, 5 and 1 days prior to sacrifice. High molecular weight polyinosinic:polycytidylic acid (polyI:C) was obtained from Sigma-Aldrich UK, and administered at a dose of 100 µg/mouse i.p. 24 h before assessment of its impact on NK cells.

Splenocytes were cultured in complete RPMI (Sigma) with penicillin streptomycin at 2 million cell per ml supplemented with 50 U/ml IL-2 or 50 ng/ml IL-15 (PeproTech) at 37°C 5% CO2 in a humidified incubator. To generate pure NK cells samples for RNA or protein extraction NK cells were enriched by negative selection using MojoSort™ Streptavidin Nanobeads (Biolegend). Dissociated lymphocytes were incubated with biotinylated anti CD3, F4/80, CD19, Ly6G, CD8, CD14, CD4 and TER-119 antibodies (biolegend) washed and resuspended in 3ml of mojo buffer (Biolegend), Streptavidin Nanobeads added and incubated for 15 min 4 °C. Tubes were placed in a magnet at RT for 5 min, supernatant collected in addition to supernatant from a wash of the beads with mojo buffer. Enriched NK cells were resuspended in complete RPMI containing 50 ng/ml IL-15 to achieve a density of $0.1x10^5$ NK cells/ml. Post culture NK cell were sorted for greater purity by their intrinsic GFP expression on a FACSAria (BD).

Transduction

293T cells used for transfection were kindly provided by Dr. Artavanis-Tsakonas (Imperial College London). Cells were grown in DMEM supplemented with 5% foetal bovine serum. Cells were grown at 37°C in a 5% CO2 incubator. 293T cells were transfected with VSV-G pseudotyped lentiviral vector SIN18-RhMLV-Cppt-2E (Tran

and Kung, 2007) containing *GFP or Ncr1* in conjunction with packing plasmid 8.2vpr and VSVG containing plasmid at a ratio of 1:1:1 using TransIT®-293 (Mirus). 48h and 72h later viral particle containing media was harvested, clarified and spun onto T100B RetroNectin® Recombinant Human Fibronectin Fragment (Takara) coated 24 well plates at 2000xG for 1h at 37 °C then allowed to adhere for a further 4h at 37 °C in a 5% CO_2 incubator. Viral particle containing supernatant was removed and replaced with $6x10^5$ NK cells that had been cultured for 3 days in 1000U/ml IL2. NK cells were left on this plate over night before being transferred onto a second plate coated with the 72h post transfection 293T cell supernatant. 3 days later NK cells were assessed for TRAIL and NKp46 expression by flow cytometry.

RNA isolation and quantitative RT-PCR

RNA was extracted using Qiagen's RNeasy kit (Hilden, Germany) and reverse transcribed into cDNA with High capacity cDNA RT kit (ThermoFisher Scientific). An amplification step was performed using the TaqMan PreAmp Master Mix Kit (Applied Biosystems). Quantitative real time PCR was carried out using the TaqMan system (Applied Biosystems), all values were normalized to GAPDH expression.

Western Blotting

Total cell lysate was prepared by using Digitonin lysis buffer (1 % Digitonin, 50 mM Tris pH 7.4, 150 mM NaCl). Cells at a concentration of $2x10^7$ cells/ml were lysed for 20 min on ice and nuclei removed by centrifugation. Cell lysates were resolved under denaturing conditions, transferred to PVDF membrane and immunoblotted with either anti-TRAIL (AF1121, R&D Systems), or anti-actin (SC-47778, Santa Cruz) antibodies. An XRS+ gel doc system and Image Lab software (BioRad) was used to capture and analyse images.

ImageStream

Primary splenocytes were stained as per the flow cytometry protocol with the exception of Zombie NIRTM (Biolegend) that was used as the viability dye. Samples were run on ImageStreamX Mark II (Amnis, USA) and analysed using IDEAS® software.

RESOURCE TABLE

REAGENT or RESOURCE	SOURCE	IDENTIFIER
Antibodies		
V450 / BV421 / APC-Cy7 / BV605 /conjugated anti mouse CD3 – clone 17A2	Biolegend / BD Biosciences	561389 / 100228 / 100221 / 100237
FITC conjugated anti mouse CD3 – clone 145-2C11	Biolegend	100305
Biotin / APC / APC-Cy7 / FITC / PE-Cy7 / BV605 conjugated anti mouse CD45 – clone 30-F11	Biolegend	103103 / 103111 / 103115 / 103107 / 103113 / 103139
Biotin conjugated anti mouse CD69 – clone H1.2F3	Biolegend	104503
APC conjugated anti mouse CD49a – clone HMα1	Biolegend	142605
PE conjugated anti mouse CD49a – clone Ha31/8	BD Biosciences	562115
PE-CF594 / APC conjugated anti mouse CD49b – clone DX5	BD Biosciences / eBioscience	562453 / 17-5971-82
eFluor 660 conjugated anti mouse Eomes – clone Dan11mag	eBioscience	50-4875-80
PE conjugated anti mouse IFNγR – clone 2E2	eBioscience	12-1191-82
PE-Cy7 / Alexa Fluor 488 / PerCP-Cy5.5 conjugated anti mouse NK1.1 - clone PK136	Biolegend / BD Biosciences	108713 / 108717 / 551114
APC / FITC conjugated anti mouse NKp46 – clone 29A1.4	Biolegend / eBiosciences	137607 / 11-3351-80
Biotin / PE conjugated anti mouse TRAIL – clone N2B2	Biolegend / eBioscience	109303 / 12-5951-81
B-Actin Antibody (C4) – clone sc-47778	Santa Cruz Biotechnology	Sc-47778
Mouse TRAIL/TNFSF10 Antibody – clone AF1121	R&D Systems	AF1121
Chemicals, Peptides, and Recombinant Proteins		
Recombinant murine IL-2	Peprotech	212-12

Recombinant murine IL-15	Peprotech	210-15
polyinosinic:polycytidylic acid (polyI:C)	Sigma-Aldrich	P1530-100MG
α-Galactosyl Ceramide	Avanti Polar Lipids	867000P
Critical Commercial Assays		
BD Cytofix/Cytoperm™ Fixation/Permeabilization Solution Kit	BD Biosciences	554714
eBioscience ™ Foxp3 / Transcription Factor Staining Buffer Set	ThermoFisher Scientific	00-5523-00
MojoSort™ Streptavidin Nanobeads	Biolegend	480016
RNeasy Mini kit	Qiagen	74104
High capacity cDNA RT kit	ThermoFisher Scientific	4368814
TaqMan PreAmp Master Mix Kit	ThermoFisher Scientific	4384267
Experimental Models: Organisms/Strains		
Ncr1$^{+/gfp}$, *Ncr1*$^{gfp/gfp}$ mouse strains	(Gazit et al., 2006) / The Jackson Laboratory	022739
